# Mutation analysis of TNP1 gene in infertile men with varicocele

**Published:** 2014-04

**Authors:** Mohammad Mehdi Heidari, Mehri Khatami, Ali Reza Talebi, Fahime Moezzi

**Affiliations:** 1 *Department of Biology, Faculty of Sciences, Yazd University, Yazd, Iran.*; 2 *Department of Anatomy, Research and Clinical Center for Infertility, Shahid Sadughi University of Medical Sciences, Yazd, Iran.*

**Keywords:** *Varicocele*, *TNP1 gene*, *Infertility*, *Single-Stranded Conformational Polymorphism*

## Abstract

**Background:** Varicocele is associated with the failure of ipsilateral testicular growth and development, and the symptoms of pain and reduced fertility. The highly condensed structure of the sperm nuclear chromatin is provided by proper expression of Transition Nuclear Protein (TNP) genes, so any dysregulational expression of these genes results in abnormal spermatogenesis and infertility.

**Objective:** The aim of present study was to assess the association between TNP1 mutations and varicocele in Iranian infertile men.

**Materials and Methods: **Analysis of association between TNP1 gene mutation and varicocele phenotype was performed using PCR and Single-Stranded Conformational Polymorphism technique and DNA sequencing in 82 varicocele infertile men and 80 control subjects.

**Results: **Sequence analysis was identified one variant in this gene that found in 15 infertile men and was absent in control group. This variant was a single nucleotide polymorphism that were identified in the intron region of this gene at position g.IVS1+75T>C.

**Conclusion:** The effect of this nucleotide substitution in intronic region of the TNP1 gene and their role on expression remains to be determined.

## Introduction

Varicocele is a common abnormality with the failure of ipsilateral testicular growth and development, and the symptoms of pain and reduced fertility (1). This physical abnormality is the main cause of primary and secondary male infertility worldwide (2). The clinical varicocele is present in 11.7% of men with normal semen analysis and in 25.4-81% of men with abnormal semen (1, 3). Although the exact pathophysiology of varicoceles is not known, but the proposed mechanism include reflex of warm abdominal blood through incompetent valves of the spermatic veins and therefore cause an abnormal dilation of the testicular veins within the pampiniform plexus (4, 5). 

In the other hand, the cause of impaired spermatogenesis includes oxygen deprivation, poor venous drainage that leads to impaired drainage of gonadotoxins from the testis, and increased oxidants within the semen (6). The integrity of sperm DNA is an essential factor for normal transmission of genetic materials during the process of fertilization as well as embryo development (7). Recently, it has been reported that varicocele influences the sperm chromatin condition, as well as sperm concentration and motility (8, 9). Some genes are expressed in both germ and somatic cells, but others are exclusively expressed in germ cells and any disruption to the regular expression of these genes may lead to abnormal spermatogenesis (10). 

During spermatogenesis, the sperm nucleus undergoes a marked rearrangement which involves the removal of histones and their replacement by various nuclear proteins, including highly positively charged protamines (7, 11, 12). The replacement of histones and the deposition of protamines are supported by different nuclear proteins, including the transition nuclear proteins (TPs) for major remodeling of the chromatin (13). Structure of the sperm nuclear chromatin becomes stable following transformation to DNA-protamine complex (7, 14). One of the predominant functions proposed for these important sperm proteins is preserving DNA integrity in the sperm head by preventing attack from exogenous or endogenous agents (15). 

Talebi *et al* showed that varicocele samples contain a higher proportion of spermatozoa with abnormal DNA and immature chromatin than those from fertile men as well as infertile men without varicocele (9). Human have two TNP and two PRM genes. The nucleoprotein genes PRM1, PRM2 and TNP2 are closely linked in a stretch of DNA, 13-15 kb long on human chromosome 16p13.3 that are categorized in protamine gene family and the TNP1 gene is located at 2q35 position (16). Mutation in TNP and PRM genes has been reported to be associated with DNA damage in azoospermia patients, but their association with varicocele patients have not exactly reported yet (16, 17). Since one of the important genes that play role in chromatin condensation is TNP that are replaced by the PRMs during chromatin condensation in the mature sperm nucleus, probably TNP defected proteins cause abnormal condensation of sperm chromatin, increases sperm DNA strand breaks and immobility of spermatozoa that can lead to male infertility (17). 

The aim of present study was to investigate the association of TNP1 mutations with varicocele-associated infertility patients.

## Materials and methods

**Patients**

This study is a cross sectional study. Blood samples were collected by venopuncture in EDTA-coated tubes for DNA extraction and genotypic analysis from 2009-2012 in the Research and Clinical Center for Infertility in Yazd. 

This study included eighty two Iranian infertile men with varicocele and eighty healthy donors with proven fertility and normal spermogram at the time of study, as control group. The varicocele diagnosis was made, by the same urologist, for the patients in standing position and via scrotal palpation in a temperature controlled room (23^o^C). Semen analysis was performed according to WHO criteria (18). Collected samples were stored at -20^o^C until analysis. This study was approved by ethical committee of Biology Department of Yazd University. All of the participants were informed of the aims of the study and gave their informed consents to the genetic analysis 

**DNA isolation and PCR amplification**

**Figure 1:**
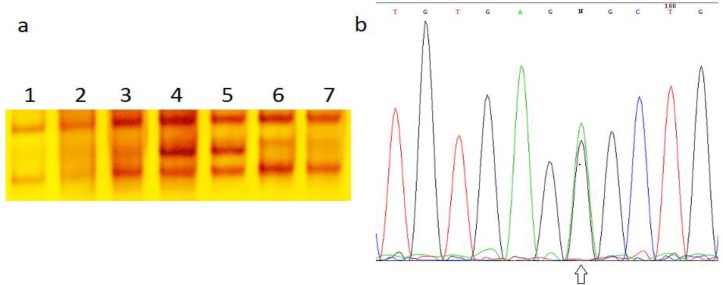
a) Identification of a Heterozygotic mutation in infertile men with varicocele by SSCP and sequencing. Lane 4 and 5, a band shift belong to g.IVS1+75T>C mutation. Lane 1, wild type.b) Sequencing result revealed g.IVS1+75T>C mutation

DNA was isolated from 200 μl of blood by a DNA extraction kit (CinnaClone, Tehran, Iran). Primer sets were designed and optimized with Gene Runner version 3.05 (Hastings Software Inc. Hastings, NY, USA, http://www. generunner.com) to amplify the two exons and intron of TNP1 gene (F:5′-CCCTCATTTTGGC AGAACTTAC-3′ and R:5′-GTTGCTGCTTGGT GCTGTGTG-3′) (Figure 1). 

Touchdown polymerase chain reaction (PCR) techniques were used and cycling condition used for the amplification was as follows: denaturation for 30 s at 94^o^C, annealing for 50s at 68^o^C, 67^o^C, 66^o^C, 65^o^C, 64^o^C, 63^o^C (two cycles for each temperature) and at 55^o^C for 22 cycles and extension for 50s at 72^o^C (final extension for 5 min). PCR of each sample was set in a 0.5 ml tube using 100 ng of total DNA, 10 pM of each primer, 200 µM of dNTPs, 1X PCR buffer containing 2.5 mM MgCl2 and 1 U Taq DNA polymerase (Roche Diagnostics, Mannheim, Germany). Using these primers, we were able to amplify fragments of 482 base pairs of the TNP1 gene.

**Single strand conformation** **polymorphism and sequence analysis of** ***TNP1*** **gene**

Single strand conformation polymorphism (SSCP) was used to screen all the samples before sending them for sequencing. In a PCR tube, 9μl PCR product and 7μl loading dye were taken. After proper mixing all samples were denatured in a thermocycler at 94^o^C for 5 min. Denatured PCR product was immediately transferred into ice to prevent renaturation and then loaded onto 8% polyacrylamide gel and electrophoresed at 130 V for 21 h. 

Gels were stained with silver to reveal the bands of single strand DNA. Various band patterns of the amplified PCR products were marked and scored. The typical gene variants got sequenced from a commercial agency (Macrogene Seoul, South Korea). The online ClustalW2 (http://www.ebi.ac.uk/tools/msa/clustalw2/) multiple sequence alignment software and Blast analysis was used to find the percent homology of the sequences that has been obtained in the study and with all other sequences of the other species.

**Statistical analysis**

Fisher’s exact probability test was used to examine the association between two groups. Values of p<0.05 were regarded as statistically significant. Statistical analysis was performed using the GraphPad Prism software (GraphPad Software, Inc. USA).

## Results

Among the total number of 162 subjects, which included 82 infertile men with varicocele and 80 control group, patients with varicocele were in 3 grade: a) Grade I (n=11); b) Grade II (n=34); c) Grade III (n=37). The mean age of infertile men with varicocele and normal controls was 28.1±5.69, and 29.4±6.12 (Mean±SD) respectively and were the groups match to each other. Single Strand Conformational polymorphism (SSCP) analyses were carried out on a total of 82 varicocele men and 80 healthy controls. Normal controls of the same ethnicity were also genotyped to establish the frequency of mutations. 

On SSCP analysis, a single band shift represents a nucleotide substitution. DNA fragments showing abnormal banding patterns on SSCP analysis were sequenced for the identification of exact mutations (Figure 1). Sequence analysis identified one variant at position g.IVS1+75T>C that found in 15 infertile subjects and was absent from the control group. This variant was a single nucleotide polymorphism that was identified in the intron region of this gene.

**Figure 2:**
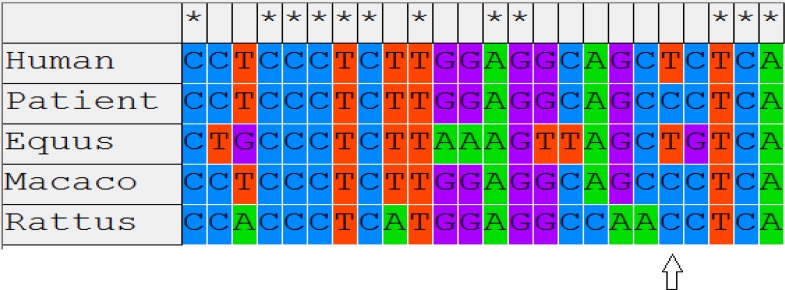
Multiple sequence alignment of g.IVS1+75T>C mutation in TNP1 gene of human with other species and the arrow (*) indicate the sites of this mutation

## Discussion

Infertility is considered as a major public health problem, because it affects about 15% of reproductively-aged couples, and male factor infertility plays a role in approximately 50% of infertile couples, that among this, varicocele-associated infertile men are more than others (19, 20). The exact association between reduced male fertility and varicocele is unknown, perhaps the accumulation of blood cause the testes temperature to be higher and so sperm production will be affected; or the pooled blood in the varicocele with higher hormonal contents may alter spermatogenesis (21). The DNA of human sperm is highly condensed in the sperm head by both PRM1 and PRM2 (22). 

Varicocele is associated with increased sperm DNA damage, and this sperm pathology may be secondary to varicocele-mediated oxidative stress. However, the mechanism by which temperature affects spermatogenesis is not clearly understood (1, 23). During spermiogenesis, sperm protamines replace somatic cell histones in a multi-step process. In the first step, in round spermatids the replacement of the both histones and non-histones proteins occurs with the transition nuclear proteins (TNPs) and then in subsequent step, in elongating spermatids, the protamines replace TNP1 and TNP2 (24, 25). Over the last decade, a number of reports have suggested a relationship between abnormal nucleoprotein expression and single nucleotide polymorphisms in protamines and TNPs genes with low male fertility (16, 17, 22, 24, 26, 27). In our research, we detected a novel nucleic acid substitution in intronic region of TNP1 gene (g.IVS1+75T>C). 

This mutation was found in 15 infertile subjects and was absent from the control group and is moderately conserved during the evolution. The amino acid sequence of TNP1 is highly conserved in different mammal species (28). De Yebra *et al* performed a preliminary mutational analysis of protamine genes in four patients with markedly altered P1/P2 ratios while no mutation was identified in protamine genes (29). Schlicker *et al* also screened 36 infertile patients with chromatin anomalies, but they failed to identify any mutation in the gene encoding P1, P2 or TP1, suggesting that altered P1 and P2 expression may have other underlying mechanisms (30). 

In contrast to these studies, Aoki *et al* screened a large patient population with known abnormal protamine ratios. They identified fifteen SNPs (three SNPs in P1, seven in P2, two in TP1 and three in TP2); however, the frequencies of these SNPs were similar to infertile individuals without protamine deficiency (31). The investigated polymorphism shows a significant difference in the allele frequencies in patients with varicocele when compared to controls (p<10^-4^). These findings indicate that this SNP may affects intron splicing in this gene and may cause human male infertility in varicocele patients, but the exact role of this alteration is still controversial.

## Footenote

***This article extracted from M.Sc. thesis (Fahime Moezzi)***

## References

[1] Jungwirth A, Giwercman A, Tournaye H, Diemer T, Kopa Z, Dohle G (2012). European Association of Urology guidelines on Male Infertility: the 2012 update. Eur Urol.

[2] Razi M, Sadrkhanloo RA, Malekinejad H, Sarrafzadeh-Rezaei F (2012). Testicular biohistochemical alterations following experimental varicocele in rats. Iran J Reprod Med.

[3] Cassidy D, Jarvi K, Grober E, Lo K (2012). Varicocele surgery or embolization: Which is better?. Can Urol Assoc J.

[4] Jarow JP (2001). Effects of varicocele on male fertility. Hum Reprod Update.

[5] Smith R, Kaune H, Parodi D, Madariaga M, Rios R, Morales I (2006). Increased sperm DNA damage in patients with varicocele: relationship with seminal oxidative stress. Hum Reprod.

[6] Heidari MM, Khatami M, Talebi AR (2012). The POLG Gene Polymorphism in Iranian Varicocele-Associated Infertility Patients. Iran J Basic Med Sci.

[7] Khalili MA, Aghaie-Maybodi F, Anvari M, Talebi AR (2006). Sperm nuclear DNA in ejaculates of fertile and infertile men: correlation with semen parameters. Urol J.

[8] Fuse H, Akashi T, Mizuno I, Nozaki T, Watanabe A (2006). Postoperative changes of sperm chromatin heterogeneity, using acridine orange staining, in varicocele patients. Arch Androl.

[9] Talebi AR, Moein MR, Tabibnejad N, Ghasemzadeh J (2008). Effect of varicocele on chromatin condensation and DNA integrity of ejaculated spermatozoa using cytochemical tests. Andrologia.

[10] Hassun filho P, Cedenho A, Lima S, Ortiz V, Srougi M (2005). Single nucleotide polymorphisms of the heat shock protein 90 gene in varicocele-associated infertility. Offic J Braz Soc Urol.

[11] Panigrahi SK, Yadav BR (2010). Polymorphism in TNP-1 gene of Murrah buffalo bulls. Afr J Biotechnol.

[12] Lewis JD, Saperas N, Song Y, Zamora MJ, Chiva M, Ausio J (2004). Histone H1 and the origin of protamines. Proc Natl Acad Sci USA.

[13] Mylonis I, Drosou V, Brancorsini S, Nikolakaki E, Sassone-Corsi P, Giannakouros T (2004). Temporal association of protamine 1 with the inner nuclear membrane protein lamin B receptor during spermiogenesis. J Biol Chem.

[14] Youssry M, Ozmen B, Orief Y, Zohni Kh, Al-Hasani S (2007). Human sperm DNA damage in the context of assisted reproductive techniques. Iran J Reprod Med.

[15] Garcia-Peiro A, Martinez-Heredia J, Oliver-Bonet M, Abad C, Amengual MJ, Navarro J (2011). Protamine 1 to protamine 2 ratio correlates with dynamic aspects of DNA fragmentation in human sperm. Fertil Steril.

[16] Miyagawa Y, Nishimura H, Tsujimura A, Matsuoka Y, Matsumiya K, Okuyama A (2005). Single- nucleotide polymorphisms and mutation analyses of the TNP1 and TNP2 genes of fertile and infertile human male populations. J Androl.

[17] Siasi E, Aleyasin A, Mowla J, Sahebkashaf H (2012). Association study of six SNPs in PRM1, PRM2 and TNP2 genes in iranian infertile men with idiopathic azoospermia. Iran J Reprod Med.

[18] (2001). Laboratory manual of the WHO for the examination of human semen sperm-cervical mucus interaction. Ann Ist Super Sanita.

[19] mirzargar MA, Yavangi M, Basiri A, Hosseini Moghaddam SM, Babbolhavaeji H, Amirzargar N (2012). Comparison of recombinant human follicle stimulating hormone (rhFSH), human chorionic gonadotropin (HCG) and human menopausal gonadotropin (HMG) on semen parameters after varicocelectomy: a randomized clinical trial. Iran J Reprod Med.

[20] Agarwal A, Said TM (2003). Role of sperm chromatin abnormalities and DNA damage in male infertility. Hum Reprod Update.

[21] Al-Naser M, Khori F, Kaabneh A, Shunaigat A (2012). Outcome of Varicocele Surgery and Infertility at Prince Hussein Urology Center. Royal Med Serv J.

[22] Imken L, Rouba H, El Houate B, Louanjli N, Barakat A, Chafik A (2009). Mutations in the protamine locus: association with spermatogenic failure?. Mol Hum Reprod.

[23] Miyaoka R, Esteves SC (2012). A critical appraisal on the role of varicocele in male infertility. Adv Urol.

[24] Aoki VW, Liu L, Carrell DT (2005). Identification and evaluation of a novel sperm protamine abnormality in a population of infertile males. Hum Reprod.

[25] Steger K, Pauls K, Klonisch T, Franke FE, Bergmann M (2000). Expression of protamine-1 and -2 mRNA during human spermiogenesis. Mol Hum Reprod.

[26] Tanaka H, Miyagawa Y, Tsujimura A, Matsumiya K, Okuyama A, Nishimune Y (2003). Single nucleotide polymorphisms in the protamine-1 and -2 genes of fertile and infertile human male populations. Mol Hum Reprod.

[27] Salamian A, Ghaedi K, Razavi S, Tavalaee M, Tanhaei S (2008). Single nucleotide polymorphism analysis of protamine genes in infertile men. Int J Fertil Steril.

[28] Kremling H, Luerssen H, Adham IM, Klemm U, Tsaousidou S, Engel W (1989). Nucleotide sequences and expression of cDNA clones for boar and bull transition protein 1 and its evolutionary conservation in mammals. Differentiation.

[29] de Yebra L, Ballesca JL, Vanrell JA, Bassas L, Oliva R (1993). Complete selective absence of protamine P2 in humans. J Biol Chem.

[30] Schlicker M, Schnulle V, Schneppel L, Vorob'ev VI, Engel W (1994). Disturbances of nuclear condensation in human spermatozoa: search for mutations in the genes for protamine 1, protamine 2 and transition protein 1. Hum Reprod.

[31] Aoki VW, Christensen GL, Atkins JF, Carrell DT (2006). Identification of novel polymorphisms in the nuclear protein genes and their relationship with human sperm protamine deficiency and severe male infertility. Fertil Steril.

